# Post‐conflict disaster governance in Nepal: one‐door policy, multiple‐window practice

**DOI:** 10.1111/disa.12455

**Published:** 2021-10-08

**Authors:** Samantha Melis

**Affiliations:** ^1^ Researcher, International Institute of Social Studies Erasmus University Rotterdam The Netherlands

**Keywords:** disaster response, disaster risk reduction (DRR), earthquake, governance, humanitarian aid, Nepal, post conflict, state‐building

## Abstract

The response to the earthquakes in Nepal on 25 April and 12 May 2015 was as overwhelming as the magnitude of the events themselves. Tensions between the humanitarian imperative and the post‐conflict state‐building agenda soon became evident. Many actors offered support by creatively complying with the state's approach, whereas others bypassed official channels completely. In post‐conflict settings such as Nepal, the situation is especially complicated because of the contradiction between policies underscoring the importance of the state in the response and the reality of the fragility of the state, which often leads to the significant involvement of aid organisations. The post‐conflict political landscape of Nepal shaped the contours of the response, as well as how actors decided to operate within them. This paper, based on empirical findings from four months of research, contributes to a better understanding of the intricacies of the post‐conflict and post‐disaster nexus in the context of a state‐led response.

## Introduction

When devastating earthquakes struck more than 30 districts in Nepal on 25 April and 12 May 2015, almost 9,000 people died and at least 21,000 were injured. This disaster affected millions of citizens, with families losing their homes and livelihoods. It was clear that its magnitude necessitated an equally large response; the role of the Nepali security forces and many public and private initiatives have been widely praised.

However, the Nepali state needed support to respond to people's needs. The immediate influx of international humanitarian agencies overwhelmed national and local governance structures. Coordination and cooperation between the different actors involved became increasingly challenging (Boersma et al., 2016), and the response further crystallised existing governance struggles between the Nepali state and international organisations (Bhatta, 2013). The earthquakes in Nepal demonstrate, therefore, the importance of understanding collaborative disaster governance premised on state–non‐state collaboration (Tierney, 2012) in the context of the post‐conflict and post‐disaster nexus.

Previous work reveals a strong relationship between disaster and conflict (Spiegel et al., 2007), with mutually reinforcing dynamics (Nel and Righarts, 2008) highlighting the political nature of both disasters and disaster response (Olson, 2000; Pelling and Dill, 2010). Yet, little research has examined what this means specifically for post‐conflict settings, which are characterised by social change and political reform (Brinkerhoff, 2005). In Nepal, the disaster unfolded in a contested political landscape: the finalisation of the new constitution had been postponed multiple times, and local elections had not been held for 28 years. A transitional period was set in motion in 2006 by the end of 10 years of armed conflict, and, in 2015, unresolved sociopolitical challenges and continuous political reform influenced the population's vulnerability to the earthquakes and the national response.

Since disaster governance centres on the state's role (Harvey, 2009), institutional changes in post‐conflict settings pose particular challenges to aid organisations balancing the humanitarian imperative with supporting the strengthening of state institutions. Harvey (2013) argues that even when states cannot fulfil their function, humanitarians should continue to engage with them. This has become even more important after the United Nations World Humanitarian Summit, held in Istanbul, Turkey, on 23–24 May 2016, and the Grand Bargain, agreed at the gathering, as international humanitarian actors have committed to localise aid among national and local responders. The centrality of the state often creates a tension between humanitarian principles and state sovereignty (Kahn and Cunningham, 2013). In practice, various challenges arise in translating disaster governance policies into outcomes (Jones et al., 2014) and disasters can become political instruments (Warner, 2013).

In Nepal, this led to contestation over control of the response and tension between aid and state actors. Although the everyday politics of humanitarian assistance are rarely addressed in the disaster governance literature, understanding how aid and state actors balance the politics associated with strengthening the state's response and the humanitarian imperative can provide valuable insights. This is crucial given these states' often limited capacity to fulfil the roles and responsibilities defined by international disaster response policies and different political institutions' attempts to establish their legitimacy in an uncertain transitional space.

This paper aims to address two frequently overlooked questions regarding the post‐conflict and post‐disaster nexus: how do state and non‐state actors negotiate a state‐led disaster response in a contested political environment?; and how does this affect local‐level disaster governance? The paper critically analyses aid actors' space for responding to disasters in post‐conflict settings, where state‐centred disaster response policies are negotiated in practice.

## State‐building and post‐conflict emergencies

### Collaborative or contentious disaster response governance?

International involvement in post‐conflict and post‐disaster processes is based on a state‐centric perspective. However, whereas post‐conflict interventions are commonly situated within a politics and state‐building discourse, post‐disaster action suggests humanitarian, apolitical practice. When these spheres converge in collaborative, consensus‐oriented disaster governance, tensions arise.

Post‐conflict state‐building primarily focuses on strengthening weak state institutions to prevent a relapse (Doyle and Sambanis, 2000; Walter, 2004; Collier, Hoeffler, and Söderbom, 2008; Nilsson, 2008; Nathan and Toft, 2011). These state institutions are often in transition, with legitimacy and power being continuously negotiated. This period is accompanied by a large influx of aid organisations and resources. Post‐conflict and post‐disaster processes are mutually constitutive. The post‐conflict milieu of a state in transition legitimises the participation of many non‐state actors in response governance. This presents challenges when the political motives and social frameworks of state and non‐state actors diverge.

Post‐disaster periods are frequently characterised by complexity in terms of humanitarian politics and the roles of non‐state actors, especially in (post‐)conflict environments. Within international humanitarian law, humanitarian assistance revolves around sovereignty, consent, and legitimacy. The need to intervene is generally accepted in times of crisis (Bellamy, 2004). Unlike international humanitarian law's legal framework, the ‘international disaster response laws, rules and principles’ have long relied on multilateral and bilateral treaties, resolutions, declarations, and guidelines (IFRC, 2007, p. 15). While significant progress has been made in formalising international disaster response laws, when the earthquakes struck in 2015, responders were still guided by a framework that was mostly based on ‘soft law’. The humanitarian principles legitimise and guide the conduct of humanitarian and multi‐mandate organisations in ‘consented space’ and are presented as apolitical. Yet, these principles are not absolute; they depend on the setting and the actors—for instance, faith‐based organisations legitimise their actions via a religious mandate (De Cordier, 2009). Scholars have shown that the principles are frequently used instrumentally (Weiss, 1999; Leader, 2000; Hilhorst and Jansen, 2010; Salomons, 2014; Bennett, Foley, and Pantuliano, 2016; Dijkzeul and Hilhorst, 2016). Much like disaster‐affected people use a ‘victimcy’ strategy to increase their access to aid (Utas, 2005), non‐state actors deploy ‘ignorancy’, where more technical approaches are foregrounded over other motives (Hilhorst, 2016).

In practice, disaster response is negotiated through everyday politics in the humanitarian arena (Hilhorst and Jansen, 2010), with actors continuously renegotiating the roles, legitimacy, and outcomes of humanitarian aid, translating policies into practice. This is especially important in post‐conflict areas because of the involvement of many multi‐mandate organisations that, in addition to providing humanitarian assistance, also actively pursue objectives that require an overtly political and rights‐based approach to address disaster vulnerabilities (Bennett, Foley, and Pantuliano, 2016, p. 52). In disaster governance, compromises are inevitably made between state and non‐state actors on the modalities of aid (del Valle and Healy, 2013).

Under international law, the sovereign state must consent to other responders assisting in disaster response (Venturini, 2012). In fact, the power imbalance between aid organisations and the state and the resulting tensions involved in assisting the state become visible during and after a disaster. State‐centred disaster response policies often do not translate into state‐centred practices to overcome this imbalance. Humanitarian aid provided by non‐state actors is regularly directed to other non‐state actors. Western government donors direct most of their funding to multilateral agencies and nongovernmental organisations (NGOs); only 2.5 per cent of this money is channelled to states (Development Initiatives, 2016, 2018), as compared with 60 per cent to multilateral agencies and 35 per cent to NGOs (Development Initiatives, 2018). These resources strengthen NGOs' power in the humanitarian arena, where collaborative disaster governance is negotiated.

### Post‐conflict politics shaping disaster policies and practice in Nepal

Research has revealed strong post‐conflict sociopolitical challenges in Nepal in relation to disaster governance. As Harrowell and Özerdem (2018) have argued, the politics of the post‐conflict process shaped the political space for the responses to the earthquakes. Nepal's Comprehensive Peace Agreement of 2006 was followed by a decade of political reform. Nevertheless, the post‐conflict period, when the disaster unfolded, was characterised by: (i) the increasing permanence of international aid agencies within the governance landscape and accompanying tensions; (ii) enduring social marginalisation; and (iii) an unstable political environment, creating difficulties for aid actors seeking to support the Nepali state's response to the earthquakes.

First, international aid organisations and external funding have become intrinsic components of governance in Nepal, creating tensions vis‐à‐vis state power even before the earthquakes struck. Since the Comprehensive Peace Agreement was signed, foreign aid has increased in Nepal. The country received more than 39 per cent more aid per capita in the first five years after the conflict ended than during the conflict period (Ndikumana, 2016). External aid represented around 20 per cent of the national budget in 2014, and there were approximately 200 international NGOs (INGOs) and more than 40,000 NGOs working in Nepal (Government of Nepal, 2014). The disaster risk reduction agenda was also largely donor‐driven (Bulkeley and Jordan, 2012; Jones et al., 2014). Furthermore, international actors' liberal state‐building agenda created multiple power centres and strengthened the position of non‐state actors, making consensus more difficult (Bhatta, 2013). The Nepali state and society have been critical of outside influences. A strong counter discourse on the state's sovereignty is supported by mistrust of the intentions of India and other external parties (Adhikari, 2014).

Second, the continued exclusion and marginalisation of lower caste and non‐caste groups have affected negotiations between state and non‐state actors in the response. According to the *National Population and Housing Census 2011*, Nepal had 126 caste and ethnic groups (Government of Nepal, 2012). Many groups have political representation, but the high caste dominates power in Kathmandu (Lawoti and Hangen, 2013). The earthquakes disproportionately affected marginalised communities and lower castes and the politicisation of aid created conflicts within communities (Shah and Acharya, 2017). As Koirala and Macdonald (2015) underline, the earthquakes were most devastating in rural areas because of centralised politics and poor development outside of Kathmandu.

Third, the volatile political environment during Nepal's post‐conflict period affected disaster management policies and practice. After the war, political reforms safeguarding equal rights and inclusion were promised, but the process was slow and highly contested. The earthquakes injected new impetus, and the new constitution was fast‐tracked. From the end of September 2015 to late February 2016, protests erupted, and a blockade of the Indian border resulted in a fuel crisis and delays in earthquake relief and reconstruction materials, bearing directly on response and recovery activities.

Moreover, because the earthquakes occurred during the pre‐constitutional period, political structures and institutions were operating in a transitional system under an interim constitution and acts pre‐dating the conflict, such as the Local Administration Act, 2028 (1971),^1^ which encouraged local self‐governance. All of the primary leadership positions were centrally appointed rather than elected,^2^ reproducing an elitist governance structure in a decentralised fashion. This affected the response because the District Disaster Relief Committees (DDRCs) were headed by district leaders, the Chief District Officers (CDOs), who regularly changed positions, largely following national‐level political developments. Consequently, these officers' party affiliation was important in terms of their role and represented part of a larger system where the central government's control still strongly moulds decentralised governance systems.

Aid agencies confronted these challenges during the responses to the earthquakes of 2015, with the post‐conflict political environment strongly defining post‐disaster governance practices. For the state, legitimacy partly depended on service delivery; thus, controlling aid resources was important for state‐building (Stel et al., 2012; Rocha Menocal, 2013; Melis, 2018). This led to wheeling and dealing of aid between state and non‐state actors.

## Methods

This paper is based on fieldwork conducted in Gorkha (Gorkha, Laprak, and Barpak Village Development Committees (VDCs)^3^), Kathmandu, and Sindupalchok (Chautara, Thautali, and Mankha VDCs) in February–May 2017. Data were collected through 123 semi‐structured interviews^4^ and eight focus group discussions (FGDs) in Barpak, Laprak, Mankha, and Thautali. A student from Tribhuvan University assisted in negotiating with gatekeepers and acted as an interpreter. A collaboration was formed with the Institute of Crisis Management in Kathmandu to discuss the findings throughout the research period.

The interview participants were selected to ensure representation of a wide array of actors who were actively involved in the response. The INGOs, international organisations (IOs) such as donors, and NGOs were contacted directly by e‐mail or telephone. The state representatives were approached by telephone and by visiting their offices. The FGD participants were assembled by each VDC's social mobiliser, who generally facilitates community development activities. The interviews concentrated on the organisation of the response, inter‐actor coordination, roles and responsibilities, and the main challenges confronted. The FGDs utilised a participatory timeline mapping the different responders and evaluation via ranking and scoring.

The main difficulties encountered during the research related to access and participation at the VDC level and the influence of the reconstruction period on the participants' views of the response. Although the FGD participants included a mixture of different castes and ethnicities, it is possible that there was a slight bias towards the political affiliation of the social mobiliser who organised the participants. However, multiple opinions were expressed and met with respect in most FGDs. My background as a foreign Western researcher may have affected the responses positively and negatively, sometimes creating a safe space for political opinions to be voiced but also potentially establishing a barrier to trust. The student partially mitigated this limitation; she was of mixed caste, which heightened trust among lower caste participants. In addition, having a local interpreter increased access in VDCs with more uniform ethnicity and caste distributions, as was the case in Gorkha, where limited Nepali is spoken.

Since the research was conducted two years after the earthquake response, the reconstruction period may have influenced the participants' opinions. To the extent possible, I included participants who were involved in the actual response or recovery. The time that had passed may also have allowed participants to reflect on the entire post‐disaster period and observe the progression of inter‐actor relationships over time.

A thematic analysis was performed by coding the interview and FGD transcripts using NVivo software. Codes were identified through a grounded approach, allowing nodes to emerge from the data. These were then divided into the themes of actor relations, challenges, discourses, and social practices of disaster response governance. These themes are discussed below in terms of: (i) the main challenges deriving from the state‐led policies and practices; (ii) the social practices of non‐state actors navigating these challenges; and (iii) variation at the local governance level. The large number of interviews allowed for the triangulation of opinions and clustering of the data to identify more generalised issues and practices that were shared among the participants.

## Aid–state relations challenging response paradigms in a post‐conflict setting

Aid responders had diverging motives and sometimes bypassed official channels. To increase control, the government imposed a one‐door policy, meaning that all non‐state responders had to pass through state institutions. In practice, however, this policy was negotiated, reinterpreted, and sometimes circumvented, allowing the provision of assistance through multiple windows. With response resources and power mostly wielded by international aid agencies, their decisions regarding supporting the one‐door policy or bypassing state institutions through multiple windows had a profound bearing. The Nepali state preferred the strengthening of state institutions through the one‐door policy because the multiple window approach required compromises and produced varying degrees of support for the post‐disaster state‐building agenda.

This section begins by discussing the most challenging state policies and approaches for non‐state actors—namely, the one‐door policy and the blanket approach to aid distribution are examined as state tactics to control non‐state actors and manage the impact of the aid response on the state's legitimacy. Next, non‐state actors' tactics to manoeuvre within or without the state system are analysed, considering these tactics on a continuum from compliance to alienation. Lastly, discrepancies observed between different districts and VDCs are considered to explore the intricacy of the political context of the response in practice and show how aid–state relations affected local governance.

### The state challenging aid: beating around the bush

Political motives of control were laid bare in the state's response, as seen in the one‐door policy, the blanket approach to aid distribution, and humanitarian assistance restrictions. Non‐state actors questioned these motives, but the Nepali state saw these policies, approaches, and restrictions as part of the state‐building agenda, aiming to support social cohesion and the legitimacy of the Nepali state.

#### The one‐door policy: confined coordination

Although the state was open to support, controlling the multitude of non‐state actors involved in the response was difficult. Research participants representing state structures strongly critiqued INGOs' lack of transparency. The state had to balance the need for aid with the accompanying influence. The one‐door policy became a cornerstone of the state's response; by formally requiring all non‐state responders to inform and include state institutions in their response, this policy controlled disaster governance and ensured that all responders fell in line. Although non‐state actors saw it as a political measure of control, the one‐door policy was rooted in national policies pre‐dating the conflict. The Natural Calamity (Relief) Act. 2039 B.S. (1982) stipulated that the Government of Nepal could require ‘foreign nationals and agencies to take the approval of the Government of Nepal to enter into any area for any purpose affected by natural Calamity’.^5^ In the relief phase, there was confusion concerning whether the one‐door policy applied only to organisational registration and coordination or also to the distribution of aid items. As aid agencies preferred to be involved in the coordination but to maintain control over their operations and resources, the one‐door policy was contested and renegotiated in practice.

The district‐level translation of the one‐door policy was largely dependent on the CDO and took different forms over time. In some districts, aid items were stored by the state. In others, a state representative simply needed to be present. This direct control of aid items resulted in accusations of misuse by state and non‐state actors, and the practice was renegotiated. An INGO officer gave the following explanation:

*In the beginning, the government said NGOs and INGOs who come here with relief materials should distribute with [the] government's one‐door system. It should distribute in front of government representatives. The relief materials were stored for a week because of the government one‐door policy, and finally the government realised that is not possible and let us do it ourselves. […] Sometimes they said NGOs and INGOs were not transparent and so on. They said the materials and money were misused. But the money [was] not misused but stuck because of [the] lengthy process*.^6^

Handing over aid items to the state presented a dilemma because non‐state responders could not secure accountability outside of their organisation. However, the state's legitimacy partly depended on service delivery, making control over these material resources important for state‐building objectives. Other parties controlling these resources would diminish the state's power. Some CDOs insisted, therefore, that aid items be stored and distributed by the state.

The government initially accepted the opening of multiple windows by non‐state actors. Nevertheless, within a month, the one‐door policy was translated into a regulation and coordination policy requiring organisations to register and coordinate with the appropriate state institutions. But no transfer of resources to the state was required. A national state official noted that the policy aimed to ensure the equal distribution of aid: ‘If we would not have introduced the one‐door policy, there's high risk that [a] few places may receive two or three times relief support, and some places may be deprived of such support’.^7^ In addition to being an argument for the one‐door policy, equality also supported the state's blanket approach to aid distribution, wherein it would be dispersed to everyone equally instead of being targeted at specific groups.

#### The blanket approach as a social cohesion measure or elitism?

The choice between targeting the most vulnerable and providing universal coverage of humanitarian aid is often made after analysing the needs and vulnerabilities of specific groups. Both options have political consequences. The state aimed for assistance of all, with interviewed state representatives reiterating that this would protect social cohesion. In contrast, many organisations preferred a targeted approach, following the humanitarian principles and acknowledging social groups' differing needs.

A district‐level state representative argued that targeting reflects negatively on the state's role in providing assistance:

*If you go to the community with limited resources and choose the persons, it just disturbs the people's harmony. People complain: ‘you provided, but not for me’. So many complaints were registered at the CDO office. […] You must provide anything in a blanket approach. So if you have limited resources, you just choose one VDC, and you go and cover the whole [village]. When we established that policy, we avoided the complaints*.^8^

This statement reveals several reasons for the blanket approach. The state received many complaints as they were unable to provide the necessary services. As well as affecting social cohesion, this also influenced assessments of the state's legitimacy. This revealed an incompatibility between the position of the state as a service provider and the state not being able to fulfil that role. Furthermore, there were others who questioned the motivation of the state for pushing this approach.

An INGO research participant pointed out that the blanket approach was also motivated by political reasons that his organisation could not accept:

*We did not do the blanket approach. […] We will not give goods to the state or government and tell them to do it instead. That would be problematic because most of the committee were the elite bodies in the VDC, and the targeted approach means they would not be receiving anything because it would go to the vulnerable ones*.^9^

Another INGO participant further explained the politics of universal aid versus targeting:

*Targeting drove the government insane. […] Even now, studies are coming out that people left behind are from specific caste backgrounds. It is not surprising, but those are the people the state never really supported. So humanitarian logic flew in the face of that. Also, why [do] you see the government, in many areas, declaring blanket targeting? It was the only way to get [the] political compromise that local institutions made. Even if a house is standing, then still target it, because that person is probably politically connected*.^10^

The state and political parties were accused by some research participants, primarily from IOs, of preferring a blanket approach because this would ensure the inclusion of higher castes and politically affiliated households, which tended to support the elitist state. The state, however, argued that the blanket approach promoted social cohesion. This created tension between state and non‐state actors, as humanitarian objectives were seen as conflicting with the state's state‐building agenda.

#### Measures of control: compliancy as agency

Political pressure from the state to conform to its state‐building objectives during the response was also seen in various restrictions aimed at regaining control. Some restrictions specified allowing emergency aid for only one month, after which tax exemptions were revoked and registration and approval through multiple state institutions became mandatory. After six months, state institutions no longer accepted any projects designated as ‘humanitarian’. Haiti's experience as the ‘Republic of NGOs’, with the international earthquake response lingering without strong state intervention, was cited as a negative example. State officials who participated in the research saw non‐state actors as not always respecting the Nepali state and culture, whereas it became clear in discussions with INGOs that they viewed the requirement to work with local partners as politically motivated. Research participants from aid organisations noted that many Nepali NGOs were aligned with a major political party and expressed mistrust of the district authorities, which influenced the choice of the NGOs with which aid organisations were allowed to work.

The response to the earthquakes was presented as apolitical by both state and aid actors. However, this discourse and the ensuing decisions were based on political choices aimed at strengthening state support and institutions. Actors were ‘beating around the bush’ by not mentioning the political nature of the conflict and tensions that were present, providing the state with a discourse of compliance and bureaucracy supporting the implementation of political measures. Based on its mandate as a policymaker and authority, the state used the strategy of ‘compliancy’ as a type of agency, advancing political motives to control other actors. To fit within the state's compliancy, non‐state actors used multiple tactics of creative compliance and non‐compliance.

### Aid negotiating with the state: creative compliance and non‐compliance

**Figure 1:**
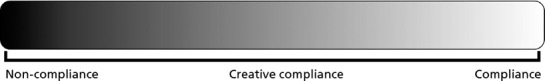
Spectrum of compliancy **Source**: author.

The role of aid actors in post‐conflict settings depends largely on the position of the sovereign state, and their compliance or non‐compliance with the state's approach affects their legitimacy as responders. Different tactics of compliance are part of a ‘spectrum of compliancy’ (see Figure 1); at one end, non‐state actors are fully compliant with the state's approach, risking humanitarian principles, whereas at the other end, non‐state actors completely bypass the state, negatively impacting on state‐building goals. In Nepal, most non‐state actors adopted different approaches and tactics to manoeuvre within state structures. On the spectrum of compliancy, these tactics are found between opposite ends and can be defined as ‘creative compliance’, which sought to balance compliance and non‐compliance, supporting the state while also adapting to fit with the aid actors' mandates.

At the non‐compliant end of the spectrum, several aid actors, particularly those who were new to Nepal or had small projects, shared the strategy of ‘perpetual non‐compliance’. An INGO worker described this strategy as an intentional response tactic:

*The operating logics were different; the humanitarians couldn't figure it out. It's funny, they were here and saying ‘this government is worse than Syria’ [and] ‘it's easier to work in Syria than it is to work here’. Okay, they are not shooting at you, but you don't have a line of NGOs waiting outside a bureaucrat's door for seven days to try to get a piece of paper signed so you can go ahead with things. The more time went on, the more that was mandatory. In the beginning, everyone was coming saying ‘general chaos’ [and] ‘the humanitarian imperative mandate’, but how long can you use that? How long can you keep on hiding certain activities [that go against the government's approach, such as direct intervention and targeting]? […] People who knew Nepal better could manage a bit better with what I call [the] ‘perpetual non‐compliance of INGOs’. We are always non‐compliant, no matter what we do. And that's intentional*.^11^

At the compliant end of the spectrum, aid actors with longstanding relationships with the state shared a vision of cooperation. A research participant from an IO stressed the importance of complying with state structures: ‘Without collaboration with the government, we can't do anything. I mean we have to constantly keep local government bodies informed about our plan. […] They suggest where to go because they have structure up to the ground level. And we have to go through that’.^12^ Although these two participants seem to express opposing ideas, in practice, there were varying degrees of compliance, even within organisations. This took shape through the adoption of different creative compliance and non‐compliance tactics, pursuing either rapprochement with or alienation from the state.

Relying on close relationships with national and local authorities was the most common tactic for responding to the disaster and strengthening organisational power to negotiate the terms of the response. Here, legitimacy as a responder was based on the role of aid agencies in supporting the state's response. Organisations that were active in Nepal before the earthquakes highlighted that this strength set them apart from newcomers and generally accorded easier access and collaboration with district authorities.

Consensus‐oriented rapprochement tactics took several forms, including using the right language. This eased the mistrust and perceived lack of transparency between the authorities and foreign organisations. Some organisations even had staff specifically dedicated to relationship building and compliance:

*We have a dedicated team with people who engage specifically with the government. We don't try and go around any of their rules and regulations. We put a lot of time and effort into actually getting MOUs [memoranda of understanding] signed—getting approval, reporting back, having meetings, and building relations with those different people*.^13^

Close collaboration with the authorities was believed to ease governance challenges such as obtaining approvals.

To deal with differences between humanitarian objectives and state policies, some organisations adopted a compromise‐based tactic operating through consortia and alliances, whereby agencies negotiated as a group. This strategy increased their power to influence response outcomes, strengthening their authority by becoming one larger unit. An example of a semi‐successful attempt is when a group of ambassadors pushed for tax exemptions for humanitarian items after the government reimposed customs duties six weeks after the disaster. Although no extension of the tax exemptions was granted, the process for applying for them became clearer. When a blockade resulted in a supply shortage, these ambassadors advocated prioritising humanitarian goods.

As a creative compliance‐based tactic to resolve differences in state and organisational approaches, the organisations adapted the state's approach to fit within their own strategies. This was especially apparent regarding the state's blanket approach, which offered universal aid coverage regardless of socioeconomic background, in contrast to non‐state actors' targeted approach, which prioritised vulnerable groups. Some organisations involved in this research agreed with the blanket approach, particularly during the relief phase; others insisted that the humanitarian imperative necessitates a targeted approach to reach the most vulnerable. However, because this targeted approach went against the state's preferences, many organisations pursued a blanket approach, but only in majorly affected or remote areas. This ‘targeted blanket approach’ was an example of creative compliance and a compromise to achieve a rapprochement with the state. When organisations did choose a targeted approach, mostly after the initial relief period, local governance structures were included to avoid tensions.

The approval mechanism presented a major challenge. To deal with this, tactics of flexibility and partial non‐compliance were utilised, ranging from starting with approval still pending to working with approval from one authority but not others. As one INGO worker stated:

*The local authorities, like the line ministries—we have permissions from those people. So we continue to work regardless of what happens and try to get the approval along the way. Most of these agencies don't have approval for a lot of things that they have already started or have to start with good intentions. They cannot wait forever. If you wait, then nothing will be done*.^14^

Another participant reported tacitly seeking approval from a certain authority:

*I'm trying to make sure the government does not think we are as large as we are, through all different sorts of ways. For example, there are two ministries to get projects approved. They have the same authority level […], so you can put half [of the] projects to the one [and] half of the projects to the other*.^15^

A related tactic was for organisations to frame their projects creatively. For instance, a religious organisation downplayed its faith‐based mandate. The government was suspicious of all faith‐based actors' motives because proselytising is illegal in the formerly Hindu country. An interviewed state representative asserted that Nepal's secularity was only established because of demands from international organisations involved in the constitutional process. Another organisation recounted deleting the word ‘humanitarian’ from a project proposal to avoid antagonising the government, which had ended the emergency period, halting the related relief operations and tax benefits. Some organisations worked together to reach solutions to duplication and approval problems, outside of the scope of the state. When two organisations discovered that they worked on similar projects in the same VDC, even when both had approval for them, they frequently collaborated to find a solution.

Notably, these creative compliance tactics required significant time and resources. Organisations and initiatives that were not well established in Nepal (and were therefore less invested in state‐building) often completely bypassed official channels, choosing to alienate themselves from the state—a non‐confrontational, contentious approach. A research participant working for a non‐registered organisation asserted that approval processes and bureaucracy took too long and that they wanted to avoid political involvement. This organisation even bypassed the local authorities. Initially, the authorities allowed direct implementation; when registration was required later, many aid organisations left. For instance, in Sindupalchok, local authorities estimated that around 200 organisations initially came, but only 103 were later registered with the DDRC.^16^

This highlights the differences between the organisations and their approaches. Generally, national NGOs felt more capable of dealing with the authorities and local political dynamics. Nepali workers in INGOs also discussed influencing local authorities as part of the response. National organisations and staff members with more local personal relations knew how to work the system. However, although they were able to manoeuvre more easily within the state system, Nepali NGOs and private organisations did not find the humanitarian coordination mechanisms inclusive. Research participants from the private sector and other new responders faced the most difficulties in navigating the state and humanitarian structures, often avoiding engaging with them. Established organisations and United Nations (UN) agencies tended to see their legitimacy as more reliant on good relationships with state institutions in a development and post‐conflict context requiring long‐term commitments. This resulted in UN agencies becoming stronger supporters of state authorities than were INGOs or NGOs, which had more space for creativity in this relationship.

The scale of the disaster, combined with the lack of clarity and political bureaucracy in state and humanitarian mechanisms, created a need for non‐state actors to manoeuvre tactically either within or without the established systems. Close relationships with the state were seen as very important, and aid agencies with a vision involving long‐term engagement in Nepal strengthened their legitimacy in the response structures. Thus, in collaborating with the state, responders adopted the tactics discussed in this section to balance humanitarian objectives with the goal of strengthening state institutions in post‐disaster governance.

### Post‐conflict aid politics: navigating differences on the ground

Gorkha and Sindupalchok were among the most earthquake‐affected districts and attracted significant international aid. Their sociopolitical contexts created differences in how the local state responded and how non‐state actors dealt with local politics in a setting of decentralised state‐building. The influx of aid affected state–society relations, and aid agencies dealt with this in different ways. Balancing state‐building with humanitarian objectives was challenging because many local authorities and politicians were heavily involved in local‐level aid governance. This empowered them to act as responders, but it also created tensions with aid actors and community members—precisely the social cohesion threats that the Nepali state wanted to avoid. The community members observed organisations coming to the VDCs through the VDC Secretaries and the political parties, which were also frequently involved in the distribution of aid. This created a politicised environment for aid at the VDC and ward level.

The one‐door policy increased the authorities' control and resources on multiple levels, ranging from national and district to VDC and ward. At the VDC and ward level, the one‐door policy was used as a measure to control the distribution of aid. Research participants recounted many examples of creative distribution: if aid was insufficient for a whole VDC or ward, it was stored locally until more items arrived or divided into smaller packages and distributed to everyone. Community members in Gorkha and Sindupalchok argued that conflicts over aid distribution were created when there were not enough items for the community. Hence, distribution outside of local systems was discouraged by local governmental stakeholders, such as local politicians, ward leaders, and social mobilisers. However, community members preferred aid distribution directly to the wards without involving local authorities, which were seen as complicit in corruption and in diverting items. The aid distribution challenges generally led to negative perceptions of the state.

In Gorkha and Sindupalchok, acts of resistance against the local authorities were observed when aid arrived. As suggested by the complaints regarding aid distribution through the local authorities, these acts mainly targeted state representatives. In one Sindupalchok VDC, the VDC office was locked by a group of protesting widows demanding a larger share of the aid. FGD participants believed the VDC Secretary to ‘always listen to powerful persons’, and many blamed politicians for corruption.^17^ There was great mistrust between the politicians and the communities. Politicians were seen as the gatekeepers to organisations and sometimes able to direct aid to their areas. In another VDC in Sindupalchok, a community leader described how politicians threatened her: ‘They would say “my party did all this, so you need to do what we are telling you”‘.^18^ Community members, in turn, accused the community leader of diverting aid. Most accusations made against the political parties concerned corruption or mismanagement, either during the aid distribution or relating to incentives received from organisations.

In Gorkha, community members' stronger ties with ward‐level representatives and social mobilisers resulted in these officials being less vulnerable to accusations, but political leaders were still viewed negatively, and aid agencies were criticised. In one VDC, the Secretary was driven out and replaced by someone with better relations with the community. Furthermore, some politicians involved in aid distribution were threatened. Even without local authorities' involvement, unequal distribution between wards caused conflicts. As mentioned above, community members preferred aid to be delivered directly to the wards and distributed equally to avoid tensions. Especially in Barpak, the first earthquake's epicentre, organisations were criticised for performing assessments and taking pictures rather than providing sufficient aid. In Gorkha and Sindupalchok, the district‐level authorities questioned the transparency of the responding organisations, and aid agencies had to negotiate their access.

Non‐state actors dealt with political parties' influence at the district and local level by either accepting or balancing political pressure, including political structures in the response, and through negotiations. In one incident, a threatened INGO pressured to support a political party leader accepted this coercion: ‘Sometimes we have to kneel down before those people. That cannot be avoided. But that cannot be more than five per cent [of the cases]‘.^19^ Yet, when another party in Sindupalchok threatened INGOs, the organisations joined together, overcoming the pressure to accept corruption. In most cases, political parties were included in the response, directly in the VDCs during aid distribution, during beneficiary identification, or as part of monitoring mechanisms.

Being transparent and open eased political pressure. As one INGO representative explained:

*[The] communication and sharing part is very important. That's the best way to deal with political parties. We don't directly deal with them, but we provide all the information through the DDRC and district offices. If there is great dissatisfaction, they request the DDRC for a meeting with all NGOs, in [the] presence of [the] CDO and NGOs, etc. […] And in that meeting, all the political parties are present. […] If there is any question regarding our spending [or] our response, we answer them*.^20^

In general, when the organisations noticed interference by political parties, they negotiated successfully with them, convincing them to let the aid activities continue.

Political influence was evident at the district and VDC level, with non‐state actors experiencing it in many different ways. Collaboration was generally regarded as easier in Sindupalchok than in Gorkha, where an organisation's reputation greatly depended on its relationship with the CDO and Local Development Officer. The research participants from aid organisations believed the Gorkha district authorities to be more politically biased towards INGOs, as compared with those in Sindupalchok. A district authority representative stressed that controlling INGOs was important because ‘[e]veryone wants their supremacy. They want to be in the spotlight. But we control them through mechanisms’.^21^ The authorities viewed the organisations as lacking transparency. A faith‐based organisation was criticised and blocked by the authorities for supposedly engaging in religious activities. After publishing a report containing a critique of the state's response, an IO was banned and a collaborating INGO's reputation was damaged. This set a precedent for others to be more careful. A Gorkha‐based INGO worker underlined: ‘It puts pressure on us because today they are saying it to them, but tomorrow it may happen to us’.^22^ A district authority confirmed that organisations had to be vigilant in Gorkha: ‘They knew that Gorkha is a Maoist‐prone area, so if they did more irregularities they would be punished by the public level’.^23^ In collaboration with some INGOs, the Gorkha authorities and political parties created NGO guidelines and a code of conduct, including monitoring mechanisms. The authorities were proud of the ‘Gorkha Model’ as an example of a well‐coordinated response, but the organisations saw their space restricted and felt pressure from local political parties in the DDRC, which were seen as directing aid towards their constituencies.

## Conclusion

The response to the earthquakes in Nepal on 25 April and 12 May 2015 was situated within a larger shift in international humanitarian policies and practices. The UN World Humanitarian Summit and the Grand Bargain of 2016 argued for a more inclusive and participatory approach to aid governance, prioritising national and local actors. The present findings contribute to understanding the challenges of a locally led response in the post‐conflict and post‐disaster nexus, where compromises are made in aid negotiations between state and non‐state actors.

When the disaster unfolded in Nepal, the post‐conflict context was characterised by a volatile political environment and a large influx of aid organisations. The Nepali state therefore became increasingly controlling during the response, using ‘compliancy’ to regain power. The tension between state‐building and humanitarian objectives was primarily seen in the state's blanket approach and one‐door policy. Although the earthquakes disproportionately affected marginalised groups, the blanket approach included higher classes, which generally supported elite state officials. This ran counter to the humanitarian imperative of targeting the most vulnerable, given limited resources. Yet, in the state's view, the blanket approach was important to preserve social cohesion, and the one‐door policy would empower and strengthen state authorities' role in the response. Tensions were exposed when bureaucratic processes were slow or when state officials were thought to be diverting aid. To balance different objectives and accommodate the state's response, non‐state actors made compromises with the state, using tactics of ‘creative compliance’ that ranged from rapprochement to alienation.

Tensions did not manifest only at the national level. The decentralised nature of response responsibilities affected how non‐state actors operated at the local level, as well as the impact of the influx of aid on society–state relations. The resources coming to the VDCs and wards were sometimes insufficient for all community members, and the involvement of local politicians in aid distribution created local‐level conflict. For the communities, this generally resulted in negative perceptions of the state and its legitimacy.

These cases highlight the challenge of supporting a decentralised response, which resulted in the politicisation of aid at the local level and complicated collaborative governance with non‐state actors. In Gorkha and Sindupalchok, state and non‐state actors exhibited mutual mistrust regarding transparency, influenced by the local personalities in charge. To overcome these difficulties, aid agencies accepted a certain degree of political influence to negotiate community access. Further research is needed to explore these relations of mistrust more systematically, as they also might prove important in other humanitarian settings.

In post‐conflict environments such as Nepal, integrating humanitarian and state‐building objectives is a primary goal of long‐term disaster governance. Although this research has shown that all actors make compromises in a consensus‐oriented approach to disaster response, misunderstandings surrounding underlying motives and politics persist. This encourages contentious aid tactics and negatively affects responders' legitimacy. These problems must be overcome to enable a response that is more locally led, internationally supported, and collaborative.

## Acknowledgements

This paper was made possible by a VICI grant of the Netherlands Organisation for Scientific Research (number: 453‐14‐013). The author would like to thank Prakriti Thapa and Dr Ram Thapaliya of the Institute of Crisis Management Studies in Kathmandu for their support in Nepal.

## Data availability statement

Research data are not shared.

## Endnotes

1 See https://www.lawcommission.gov.np/en/archives/category/documents/prevailing-law/statutes-acts/local-administration-act-2028-1971 (last accessed on 4 August 2021).

2 Under this system, the Chief District Officer (CDO) was appointed by the Ministry of Home Affairs to head the District Administration Office and acted as a representative of the government on the district level. The Ministry of Federal Affairs and Local Development appointed the Local Development Officer (LDO) on the district level to lead the District Development Committee, and the Secretary of the Village Development Committee (VDC) on the village level.

3 VDCs were administrative units under the district level, comparable to villages. A VDC was further divided into several Wards.

4 Interviews were conducted with 34 state representatives (3 national and 31 district and VDC level), 33 INGO representatives, 20 IO representatives (including United Nations agencies and donors), 12 NGO representatives, 8 security force officials, 7 private initiative or organisation representatives, 6 community members, 2 research institution representatives, and 1 journalist.

5 See https://www.lawcommission.gov.np/en/wp-content/uploads/2018/10/natural-calamity-relief-act-2039-1942.pdf (last accessed on 4 August 2021).

6 Author interview with an INGO representative, Chautara, Sindupalchok, 12 February 2017 (INGO6).

7 Author interview with a state representative, Kathmandu, 14 March 2017 (GOV22).

8 Author interview with a district state representative, Chautara, Sindupalchok, 13 February 2017 (GOV2).

9 Author interview with an INGO representative, Chautara, Sindupalchok, 16 February 2017 (INGO5).

10 Author interview with an INGO representative, Kathmandu, 9 May 2017 (INGO8).

11 Author interview with an INGO representative, Kathmandu, 9 May 2017 (INGO8).

12 Author interview with an IO representative, Chautara, Sindupalchok, 14 February 2017 (IO1).

13 Author interview with an INGO representative, Kathmandu, 13 February 2017 (INGO1).

14 Author interview with an INGO representative, Kathmandu, 9 May 2017 (INGO16).

15 Author interview with an INGO representative, Kathmandu, 9 May 2017 (INGO8).

16 Author interview with a state representative, Chautara, Sindupalchok, 13 February 2017 (GOV2).

17 Focus group discussion, Sindupalchok, 29 June 2017.

18 Author interview with a social mobiliser, Sindupalchok, 26 June 2017 (GOV 11).

19 Author interview with an INGO representative, Kathmandu, 9 May 2017 (INGO16).

20 Author interview with an INGO representative, Kathmandu, 9 May 2017 (INGO16).

21 Author interview with a local government representative, Gorkha, Gorkha, 11 April 2017 (GOV28).

22 Author interview with an INGO representative, Gorkha, Gorkha, 12 April 2017 (INGO21).

23 Author interview with a local government representative, Gorkha, Gorkha, 9 April 2017 (GOV6).
